# Kinetics of Depth Jumps Performed by Female and Male National Collegiate Athletics Association Basketball Athletes and Young Adults

**DOI:** 10.3390/jfmk8030108

**Published:** 2023-07-29

**Authors:** Talin Louder, Brennan J. Thompson, Alex Woster, Eadric Bressel

**Affiliations:** 1 Department of Kinesiology and Health Science, Utah State University, Logan, UT 84322, USA; brennan.thompson@usu.edu (B.J.T.); eadric.bressel@usu.edu (E.B.); 2 Dennis G. Dolny Movement Research Clinic, Sorenson Legacy Foundation Center for Clinical Excellence, Utah State University, Logan, UT 84322, USA

**Keywords:** plyometric, reactive strength index, agility, injury

## Abstract

The depth jump (DJ) is commonly used to evaluate athletic ability, and has further application in rehabilitation and injury prevention. There is limited research exploring sex-based differences in DJ ground reaction force (GRF) measures. This study aimed to evaluate for sex-based differences in DJ GRF measures and determine sample size thresholds for binary classification of sex. Forty-seven participants from mixed-sex samples of NCAA athletes and young adults performed DJs from various drop heights. Force platform dynamometry and 2-dimensional videography were used to estimate GRF measures. Three-way mixed analysis of variance was used to evaluate main effects and interactions. Receiver operating characteristic (ROC) curve analysis was used to evaluate the combined sensitivity and specificity of dependent measures to sex. Results revealed that reactive strength index scores and rebound jump heights were greater in males than females (*p* < 0.001). Additionally, young adult females showed greater peak force reduction than young adult males (*p* = 0.002). ROC curve analysis revealed mixed results that appeared to be influenced by population characteristics and drop height. In conclusion, sex-based differences in DJ performance were observed, and the results of this study provide direction for future DJ investigations.

## 1. Introduction

The depth jump (DJ) is considered a high-intensity plyometric movement that primarily targets lower-extremity neuromuscular function. The DJ involves dropping down from an elevated platform onto a lower landing surface and immediately performing a maximal vertical rebound jump upon ground contact. DJs generate large magnitudes of vertical ground reaction force (GRF) and rates of GRF development (RFDs), which can potentially exceed the neuromuscular tolerance for eccentric loading of muscle–tendon units [1]. Additionally, during the initial phase of landing after the drop (e.g., 0–150 ms post-impact), DJs exhibit substantially greater RFDs compared to other commonly performed jumping and resistance training movements [2,3,4,5]. Thus, DJ performance relies on the stretch-shortening cycle (SSC) muscle action of the lower extremities due to the combination of substantial eccentric loading and rapid application of GRF during landing.

The SSC is fundamental to human movement performance, and is defined as a concentric action performed immediately following an eccentric stretch-load placed on pre-activated skeletal muscle [6,7]. Given the definition, DJs may be viewed as having greater specificity for lower-extremity SSC development when compared against other common plyometric techniques. These techniques either do not involve skeletal muscle pre-activation in advance of ground impact (e.g., the countermovement jump) or are performed with a purely concentric emphasis (e.g., the squat jump). Moreover, integration of both feedforward and feedback control enhances the neuromotor complexity of DJs relative to other common laboratory assessments of jumping ability [7].

There is growing interest in using DJs to prevent and rehabilitate anterior cruciate ligament (ACL) injuries [8,9,10,11,12,13,14,15,16]. The risk of sustaining an ACL injury in sports that involve jumping and pivoting movements is significantly higher in females versus males, with the relative risk estimated to be between 4 and 6 times higher for females [17]. As a result, there have been many investigations conducted to examine for sex-based differences in DJ biomechanics. Prior research on joint-level mechanics is extensive, with evidence suggesting that post-pubescent females tend to express greater knee valgus during the entirety of the DJ drop landing phase [18,19,20,21,22,23,24,25,26,27,28,29,30] and less knee flexion at DJ landing impact [19,31,32]. Additionally, when compared with similarly aged males, post-pubescent females are observed to favor absorption of landing impact momentum through the knee rather than the hip [25,33,34,35,36,37]. 

The existing literature has significant gaps concerning sex-based differences in DJ GRF measurements across various sport and recreational populations. Specifically, in the context of basketball, a sport known for notable disparities in lower-extremity injury rates between sexes [38], there is a need to establish comprehensive baseline data across a range of DJ drop heights, obtained through internally valid methods. It is widely accepted that post-pubescent females exhibit lower rebound jump heights (JH) and reactive strength index (RSI) scores compared to similar male sub-cohorts [18,39,40,41,42,43,44]. These disparities are believed to be influenced by fundamental differences in anthropometrics, with males biologically predisposed to carry more muscle mass relative to total body mass. Consequently, this leads to a greater ability to generate concentric impulse, leading to higher JHs [45].

Prior studies investigating sex-based differences in DJ performance have generally relied on the flight time method to estimate JH [18,39,40,41,42,43,44]. However, the utilization of this approach has raised concerns regarding known threats to internal validity [46,47]. A review of the basketball jumping literature identified measurement error in JH estimation as a notable limitation associated with the flight time method [48]. Specifically, the variability in JHs derived from flight time limits the ability to compare results between subjects and benchmark across investigations with similar DJ protocols. These concerns have led to the development of novel single-force platform methods that enable the estimation of JH from the integration of GRF time-series data [46,47]. Despite offering improved internal validity, these methods have yet to be widely adopted in the DJ literature. Incorporating these advanced approaches to establish baseline data would significantly enhance the validity and comparability of DJ JH and RSI data, thereby benefiting future research in the field.

Additionally, analyzing GRF data from DJs can provide valuable insights into various dependent measures, including peak GRF, rate of force development (RFD), lower-extremity stiffness, and peak force reduction (PFR). Previous cross-sectional studies have examined differences in peak GRF and vertical stiffness (k_v_) between males and females, but inconsistent results have been reported [20,31,42,43,44,49]. These discrepancies can be partially attributed to variations in study design, such as the use of different dropping heights across sexes, data analysis methods, and populations with varied backgrounds in athletic participation [20,31,42,43,44,49]. Considering the substantial economic and time commitment required, it is also important to carefully consider study design for longitudinal DJ investigations. Longitudinal studies often adopt a prospective cohort structure to evaluate the DJ’s ability to screen for lower-extremity injury risk, specifically non-contact ACL injury risk [50]. Furthermore, in addition to providing baseline data across sub-cohorts of males and females with varying athletic backgrounds, a cross-sectional investigation utilizing internally valid methods that encompass a range of drop heights, a comprehensive collection of GRF measures, and multiple sub-cohorts of males and females, could provide valuable guidance for the design of future longitudinal investigations. Thus, the purpose of this study was to evaluate sex-based differences in DJ GRF measures and determine sample size thresholds for binary classification of sex. To address current gaps in the literature, data was collected from DJs performed at different heights by mixed-sex samples of National Collegiate Athletic Association (NCAA) Division I basketball players and physically active young adults.

## 2. Materials and Methods

### 2.1. Participants

Twenty-one NCAA Division I basketball players and 26 physically active young adults volunteered to participate in this investigation (Table 1). Participants were recruited from a university community. To be eligible for the investigation, participants were required to be between the ages of 18 and 35 and report no recent history of lower-extremity injury or surgical intervention on the lower limbs or trunk (<12 months). NCAA basketball players were involved in a pre-season strength and conditioning program, while the sample of young adults were required to self-report engaging in moderate to vigorous physical activity for at least 3 days per week. Participants were recruited on a voluntary basis, and provided written consent after reviewing an informed consent document approved by the University’s Institutional Review Board.

### 2.2. Procedures

Participants underwent a 15 min warm-up protocol that consisted of a self-selected-pace 5 min jog, followed by dynamic exercises such as high knees, carioca, lateral shuffle, and jumping jacks. After the warm-up, participants were given a 5 min rest before proceeding to familiarization of procedures. During familiarization, a member of the research team provided a demonstration of the DJ technique, and participants were required to perform at least 5 DJs across a range of drop heights. The practice trials were monitored by a research team member with expertise in the DJ technique, who provided feedback to participants as needed.

After familiarization, reflective markers were attached to body segment endpoints as specified by the de Leva [51] anthropometric model, which took approximately 20 min. Participants then underwent a testing protocol consisting of three DJ trials at drop heights of 0.51, 0.66, and 0.81 m, with the order of conditions randomized for each participant. All three trials were performed at a given height before proceeding to the next condition. The selection of drop heights was informed by a meta-analysis [52] that reported that plyometric training interventions may include DJs performed from drop heights ranging between 0.12 and 1.10 m. Additionally, in our recent work [53], we observed that GCTs were not significantly impacted when DJs were performed from the range of drop heights included in the current investigation.

A custom-made plyometric box, measuring 0.51 m × 0.66 m × 0.81 m, was placed 0.15 m behind an in-ground tri-axial force platform (Model FP4080, Bertec Corporation, Columbus, OH, USA). During each DJ trial, participants stood on top of the box and were provided with the standard verbal cue: “Step forward off the box with your preferred foot, land with both feet hitting the ground simultaneously, and then immediately perform a maximal effort jump upwards as quickly and as high as possible.” Upon receiving the cue, participants were instructed to initiate the DJ movement with no arm motion restriction. All DJ trials were monitored in real-time by a member of the research team, and to be deemed successful, participants had to land with full foot contact on the force platform and perform the maximal effort jump upwards with minimal pause upon landing. Participants were given a one-minute rest between trials and a five-minute rest between conditions [54].

### 2.3. Data Acquisition and Analysis

A high-speed camera (300 Hz; Model EX-F1, Casio, Shibuya, Tokyo, Japan) was used to capture kinematic data during the DJ trials. Video recordings were pared to start at the onset of the movement and end when participants made full foot contact with the force platform upon landing. Videos were then digitized using the Kinovea open-source software program (version 0.8.27, www.kinovea.org). Specifically, vertical position data for all body segment endpoints were digitized, with previous studies demonstrating the reliability of this approach [55,56,57]. The resulting segment endpoint position data were filtered using a low-pass, recursive, 4th-order Butterworth filter with a cut-off frequency of 6 Hz in MATLAB^®^ (version R2019b; The Mathworks Inc., Natick, MA, USA). Segmental center of mass (CoM) position data were estimated by applying the de Leva [51] anthropometric model to filtered segment endpoint data. Vertical position data for the whole-body CoM were then estimated by taking the sum of segmental CoM position data after they were weighted according to segment mass percentages from de Leva [51]. Finally, the vertical velocity data for the whole-body CoM were estimated using the first central difference method to calculate the first derivative of the vertical whole-body CoM position data with respect to time. The maximal value of the resulting downward whole-body CoM velocity was used to estimate landing impact velocity (v_i_; m × s^−1^).

MATLAB^®^ was used to process vertical GRF data acquired from the in-ground force platform and estimate outcome measures. GRF data were filtered using a low-pass, recursive, 4th-order Butterworth filter with a 300 Hz cut-off frequency. GRF data were then pared to the duration of contact between the feet and force platform, with the timing of landing impact and rebound jump take-off defined using methods described previously [54]. GCT (s) was calculated as the time duration of pared GRF data. To estimate JH (m), a mixed-methods approach was utilized [46,58], which involved a single integration (trapezoidal rule) of GRF data to obtain an estimate of the change in vertical velocity (∆v; m×s^−1^; Equation (1)) occurring between landing impact and rebound jump take-off. Rebound jump take-off velocity (v_t-off_) was then estimated by subtracting the v_i_ derived from digitized video data from ∆v (Equation (2)). Finally, JH was calculated from v_t-off_ using a constant acceleration equation (Equation (3)). After estimating JH, the RSI was calculated as the ratio of JH to GCT for each DJ trial (Equation (4)). The trial with the highest RSI score for each DJ drop height condition was selected for further data and statistical analysis.

(1) $$∆v=\frac{\int \mathrm{GRF}-Body\;Weight}{\mathrm{Body}\;\mathrm{Mass}}$$

(2) $$v_{t\text{-}\mathrm{off}}=∆v-\left|v_{i}\right|$$

(3) $$\mathrm{JH}=(\frac{{v_{t\text{-}\mathrm{off}}}^{2}}{19.62})$$

(4) $$\mathrm{RSI}=\frac{\mathrm{JH}}{\mathrm{GCT}}$$

**Figure 1:**
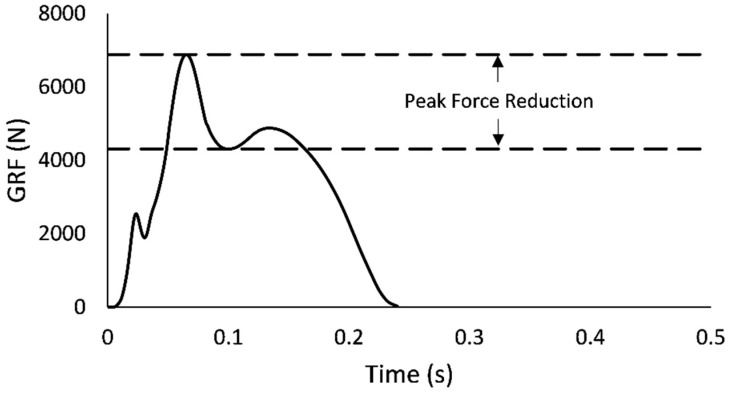
A sample vertical ground reaction force (GRF) trace with visual representation of peak force reduction (PFR) in depth jumping (DJ).

After identifying the best DJ trial at each drop height, peak GRF (N) was defined as the maximal value from pared GRF data. RFD (N × s^−1^) was calculated as the ratio of peak GRF to the time interval between the DJ landing impact and the expression of peak GRF (Equation (5)). PFR (N) was estimated by computing the difference between the peak GRF and the first successive local minimum GRF value (Figure 1). Peak GRF, RFD, and PFR were then normalized to body mass (kg) and presented in units of N × kg^−1^, N × s^−1^ × kg^−1^, and N × kg^−1^, respectively. Amortization time (Amort; s) was estimated as the time interval after landing required for ∆v to offset v_i_ (Equation (6)). k_v_ was modeled as a linear spring-mass by calculating the ratio of peak GRF to whole-body CoM displacement (Equation (7); [59]). Whole-body CoM displacement (d_CoM_) was obtained from the double integration of GRF data (Equations (8) and (9); [60]), representing the downward displacement between landing impact and the lowest CoM position achieved during the landing phase. Lastly, k_v_ was normalized to the participant’s body weight (N) and height (m), and presented in units of BW × ht^−1^ [49].

(5) $$\mathrm{RFD}=\frac{\mathrm{Peak}\;\mathrm{GRF}}{∆t}$$

(6) $$\mathrm{Amort}\;=\;t_{∆v-\left|v_{i}\right|=0}-t_{\mathrm{landing}\;\mathrm{impact}}$$

(7) $$k_{v}\;=\;\frac{\mathrm{Peak}\;\mathrm{GRF}}{d_{\mathrm{CoM}}}$$

(8) $$v=\frac{\int \mathrm{GRF}-Body\;Weight}{\mathrm{Body}\;\mathrm{Mass}}-v_{i}$$

(9) $$d_{\mathrm{CoM}}=\int v$$

### 2.4. Statistical Analysis

We conducted an a priori power analysis in G*Power (version 3.1) [61] using DJ RSI data from previously published studies [62,63]. The analysis indicated that our study had adequate statistical power to detect differences in DJ performance, as measured by the RSI (α = 0.05, 1 − *β* = 0.80, ES = 2.61, required *n* = 6). Considering the study’s design, we decided to recruit a minimum of ten subjects per group. 

All statistical analyses were performed with R programming [64] in RStudio (version 1.1.456) [65]. An alpha type I error threshold of *p* < 0.05 was used to determine statistical significance. Bivariate correlations were computed to evaluate for linear relationships between dependent measures. Significant correlations were interpreted as very weak (*r* = 0.00–0.19), weak (*r* = 0.20–0.39), moderate (*r* = 0.40–0.59), strong (*r* = 0.60–0.79), and very strong (*r* = 0.80–1.00) [66]. Three-way mixed analysis of variance (ANOVA) (drop height (0.51 m DJ × 0.66 m DJ × 0.81 m DJ) × population (NCAA Division I basketball players × young adults) × sex (males × females)) was used to evaluate for main effects and interactions. Post hoc analysis on the main effects of sex and population was performed using independent *t*-tests. Post hoc analysis on the main effects of drop height was performed using repeated measures ANOVA followed by Bonferroni-adjusted paired sample *t*-tests. Post hoc analysis on two-way interactions was performed using Bonferroni-adjusted independent *t*-tests. 

To evaluate the ability of the dependent measures to accurately classify subjects by sex, we constructed receiver operating characteristic (ROC) curves in addition to conducting ANOVA. The area under the curve (AUC) was estimated for each ROC curve, and their statistical significance was evaluated using methods from Obuchowski, Lieber, and Wians [67], with a statistical power of 0.80. We also estimated the minimum sample size required to detect statistical significance for each AUC using the methods described by Obuchowski et al. [67]. We classified AUC values as having excellent (0.90–1.00), good (0.80–0.89), fair (0.70–0.79), poor (0.60–0.69), or no discrimination (0.50–0.59) [68].

## 3. Results

### 3.1. Bivariate Correlation

Through bivariate correlation analysis, we found several notable associations between dependent measures, ranging in strength from very weak (*r* = 0.17) to very strong (*r* = 0.95; see Table 2). As expected, higher JHs (*r* = 0.82) and shorter GCTs (*r* = −0.51) had very strong and moderate associations, respectively, with greater RSI scores, given that they are component measures of the RSI. Faster RFDs (*r* = 0.36) and shorter amortization times (*r* = −0.50) had weak to moderate associations with greater RSI scores, respectively, and likely influenced the GCT component of the RSI, as evidenced by the very strong association between GCT and amortization time (*r* = 0.95). Additionally, shorter GCTs had weak to strong associations with higher peak GRF (*r* = −0.37), RFD (*r* = -0.78), and vertical stiffness (*r* = −0.20). Weak negative associations were observed between JH and both peak GRF (*r* = −0.21) and PFR (*r* = −0.24). Lastly, PFR had weak to strong positive associations with k_v_ (*r* = 0.51), peak GRF (*r* = 0.66), and RFD (*r* = 0.33).

### 3.2. ANOVA

#### 3.2.1. Interactions

No significant 3-way interactions between sex, population, and drop height were observed (*F* = 0.0–0.2, *p* = 0.661–0.945), and there were no population × drop height interactions (*F* = 0.0–0.4, *p* = 0.550–0.910) or sex × drop height interactions (*F* = 0.0–3.5, *p* = 0.065–0.919). However, significant sex × population interactions were found for GCT (*F* = 7.6, *p* = 0.007), PFR (*F* = 10.8, *p* = 0.001), and Amort (*F* = 12.1, *p* < 0.001). The results of post hoc comparisons revealed that female NCAA athletes had significantly shorter GCT (*p* = 0.020) and Amort (*p* = 0.011) compared to male NCAA athletes. Furthermore, male young adults had significantly shorter Amort compared to male NCAA athletes (*p* = 0.019), but no significant differences were found in either GCT or Amort between young adult males and females. Lastly, PFR was significantly greater for female young adults compared to male young adults (*p* = 0.002), with no significant difference in PFR observed between male and female NCAA athletes (Table 3). No significant sex × population interactions were observed for RSI scores (*F* = 3.4, *p* = 0.069), JH (*F* = 0.7, *p* = 0.389), peak GRF (*F* = 0.0, *p* = 0.899), RFD (*F* = 2.4, *p* =0.123), or k_v_ (*F* = 0.4, *p* = 0.533).

#### 3.2.2. Main Effects

ANOVA revealed a significant main effect of sex on several dependent measures, including RSI scores (*F* = 24.4, *p* < 0.001), JH (*F* = 48.6, *p* < 0.001), peak GRF (*F* = 5.1, *p* = 0.026), and PFR (*F* = 13.1, *p* < 0.001). Post hoc comparisons revealed that males had significantly greater RSI scores (*p* < 0.001) and JH (*p* < 0.001), while females had greater PFR (*p* = 0.005; Table 4). Post hoc comparisons on peak GRF revealed no significant difference between sexes (*p* = 0.792) and there was no main effect of sex on GCT (*F* = 0.1, *p* = 0.710), RFD (*F* = 3.6, *p* = 0.060), Amort (*F* = 1.2, *p* = 0.269), or k_v_ (*F*= 0.2, *p* = 0.669).

A significant main effect of population was observed for JH (*F* = 5.9, *p* = 0.016), with post hoc comparisons revealing that NCAA athletes had greater JH compared to young adults (*p* < 0.001; Table 4). No main effect of population was observed for all other dependent measures (*F* = 0.0–1.9, *p* = 0.171–0.924).

A significant main effect of drop height was observed for peak GRF (*F* = 75.2, *p* <0.001), PFR (*F* = 83.1, *p* < 0.001), RFD (*F* = 16.5, *p* < 0.001), and k_v_ (*F* = 54.0, *p* < 0.001). These results were confirmed by a repeated measures ANOVA (*F* = 16.2–71.2, *p* < 0.001) and post hoc comparisons (*p* < 0.001 for peak GRF and PFR; *p* < 0.001-0.002 for RFD; *p* < 0.001 for k_v_; Table 5). No main effect of drop height was observed for RSI scores (*F* = 0.4, *p* = 0.524), JH (*F* = 0.9, *p* = 0.355), GCT (*F* = 0.0, *p* = 0.958), or Amort (*F* = 0.2, *p* =0.664).

### 3.3. ROC Curve Analysis

The results of the ROC curve analysis are summarized in Table 6, Table 7 and Table 8. When the data were combined across populations, significant AUCs were observed for JH (0.747-0.843, *p* < 0.05) across all three drop heights and for RSI (0.732–0.743, *p* < 0.05) specific to the 0.51 and 0.81 m drop heights. A significant AUC was also observed for PFR (0.731, *p* < 0.05) specific to the 0.81 m drop height. Significant AUCs ranged from fair to good combined specificity and sensitivity (Table 6). For data corresponding to NCAA athletes, a significant AUC was observed for JH (0.980, *p* < 0.001) specific to the 0.81 m drop height, indicating excellent combined specificity and sensitivity (Table 7). For data corresponding to young adults, significant AUCs were observed for JH (0.810–0.827; *p* < 0.05) across all drop heights, for RSI (0.810; *p* < 0.05) specific to the 0.51 m drop height, and for PFR (0.804–0.821; *p* < 0.05) specific to the 0.66 and 0.81 m drop heights, indicating good combined specificity and sensitivity (Table 8).

## 4. Discussion

Using DJ GRF data from mixed-sex samples of NCAA Division I basketball players and young adults, the aim of this study was to evaluate for sex-based differences in GRF measures and determine sample size thresholds for binary classification of sex. RSI scores were similar to those reported in the prior literature [69,70]. It is important to mention that GCTs, on average, were longer in duration than what is expected of a typical fast SSC action (<250 ms), but similar to prior studies that also cued participants to prioritize both maximal jump height and minimal contact time with the ground [69,71].

Sex had a notable effect on DJ performance, with males achieving significantly higher RSI scores (+41%, Cohen’s *d* = 0.75). Higher RSI scores in males were attributed to greater JH, a pattern supported by the prior literature [18,39,40,41,42,43,44,45]. As mentioned previously, the disparity in JH across sexes is thought to be influenced by anthropometrics, such as an increased volume of muscle mass in males, which could provide an advantage in producing concentric impulse [45]. There was no main effect of sex on GCT, suggesting that RSI scores were influenced solely by differences in JH. Interestingly, however, an interaction revealed that male NCAA athletes had longer GCTs (+80 ms) compared to female NCAA athletes and slower Amort compared to both female NCAA athletes (+50 ms) and young adult males (+40 ms). The negative impact of longer GCTs on DJ performance, when assessed using the RSI, may account for the lack of a main effect of population on RSI scores, despite the observation of higher JHs in NCAA athletes (+14%) compared to young adults.

The results of the current investigation do not provide a clear explanation for prolonged GCTs in NCAA male athletes. Previous research has indicated that height and limb length may disproportionally affect jumping performance across different levels of athletic participation and between sexes [72,73,74,75,76,77]. Specifically, height and lower-extremity limb lengths in competitive male athletes have been found to associate positively with JH [73,74,76], yet these associations do not seem to hold for recreational males and females with varying levels of athletic participation [72,75,77]. In taller male athletes, height and lower-limb length may increase CoM displacement during the concentric phase of jumping, which in turn could provide additional time to accumulate the mechanical impulse that is ultimately responsible for determining JH [72,73,78]. In theory, an extended concentric impulse duration could partially explain why higher JHs were observed in male NCAA athletes at the expense of longer GCTs, yet further investigation is required to formally assess the relationship of height and limb length with GCT. 

The recent literature acknowledges the value of parametric tests for examining the statistical relationship of between-group measures [79,80,81]. However, to strengthen the evidence of group differences in these measures, it is advantageous to establish a strong dichotomy using binary classification analyses, which include the ROC curve. Further, it is important to evaluate for consistency across multiple sub-cohort samples [79]. Binary classification analyses are most often included in prospective cohort studies reporting on the ability of the DJ to screen for lower-extremity injury risk in males and females. These studies have reported significant mean differences between injured and non-injured groups for peak GRF [68,82] as well as knee mechanics in the sagittal and frontal planes [68,82,83,84,85]. However, despite their potential utility, ROC curve AUCs constructed on these same measures tend to demonstrate poor to fair discrimination (AUC = 0.60–0.70; [68,85]), with the exception of data reported by Corban et al. (AUC = 0.88–0.92; [83]). 

Although main effects of sex were observed on JH and RSI scores in the present investigation, ROC curve AUCs demonstrated fair to good JH discrimination across all drop heights in the population-collapsed and young-adult datasets (AUC = 0.73–0.84). Further, excellent JH discrimination was observed between male and female NCAA athletes for DJs performed from the 0.81 m drop height, exclusively (AUC = 0.98). RSI scores did not discriminate between male and female NCAA athletes, with good RSI discrimination (AUC = 0.81) observed between male and female young adults specific to the 0.51 m drop height. As such, it appears that differences in DJ GRF measures between sexes may be influenced by both population characteristics and drop height. The DJ literature identifies population characteristics as a confounding factor in ROC curve analyses, as there can be considerable variability in neuromuscular training history across different sub-cohorts of males and females [79,81]. 

We incorporated a range of DJ drop heights, since there is no recognized ‘standard’ height in the existing literature. Moreover, individual variations make it likely that optimal drop heights differ among persons [86,87,88]. Adding further complexity, by using a fixed drop height in DJ assessments, may not accurately represent true drop height, as it can be influenced by step-off technique [47,48]. It is suggested that optimal drop height corresponds with the maximization of either JH or RSI [89,90,91]. Longitudinal studies have demonstrated enhanced DJ performance from plyometric training programs when utilizing optimal DJ heights determined by this approach [91]. However, the impact of using sub-optimal, optimal, or supra-optimal drop heights on the effectiveness of DJ for improving athletic performance and screening for lower-extremity injury risk remains unclear, based on the current literature. 

We observed significantly greater peak GRF, RFD, PFR, and k_v_ for DJs performed at higher drop heights. Notably, these measures increased despite the absence of any changes in RSI scores or RSI component measures. Previous investigations have demonstrated that biomechanical and neuromuscular activation measures are influenced by drop height [41,92,93]. Among the measures included in the current investigation, k_v_ is a variable that may be relevant to lower-extremity injury [50,66]. For instance, inadequate or excessive development of k_v_ prior to foot impact may contribute to lower-extremity injury [94,95,96]. Indeed, the amount of musculotendinous stiffness expressed at foot impact would, theoretically, affect the relative contribution of active and passive structures in tolerating mechanical stress. An increase in passive stabilization during foot impact is thought to be a crucial factor in non-contact ACL injury, in particular [94].

While the current investigation did not find a difference in k_v_ between sexes, it may be beneficial to consider DJ conditions that challenge the regulation of k_v_. For instance, using larger drop heights may increase the mechanical demands on the sensorimotor system to control landing forces and maintain balance. As mentioned previously, the RSI and its component measures did not show significant changes when the drop height was increased to 0.81 m for the populations studied in the current investigation. This indicates that performance was maintained across drop heights, and suggests that landing impact forces remained within a safe threshold. It is also worth noting that the lowest drop height included in the current investigation (0.51 m) exceeds the drop heights previously investigated in prospective cohort designs relating to DJ injury screening [68,82,83,84,85,86,87]. As such, drop height should receive considerable attention in the design of future prospective cohort investigations. In addition to increasing the drop height, DJ intensity could also be modified using methods that challenge sensorimotor function. The dual-task paradigm or visual disruption techniques, such as stroboscopic vision, are two examples of potentially effective approaches [9,16,97,98]. Implementation of these approaches not only increases the physical demands, but could also enhance the ecological validity of the DJ. For example, these approaches require individuals to anticipate and react to landing forces while simultaneously managing attention and cognitive processing [9,16,97,98]. 

Apart from RSI scores and JH, parametric statistics revealed PFR as an additional variable that differed significantly between sexes, with greater magnitudes of PFR observed in females. Further analysis revealed that the effect of sex was due to a 2-way interaction, with significantly higher PFR in young adult females observed compared to young adult males, and with no significant difference observed between male and female NCAA athletes. The interaction was supported by ROC curve AUCs, which demonstrated good discrimination for the 0.66 and 0.81 m drop heights in young adults. Notably, ROC curve AUCs also revealed a fair discrimination of PFR for sex for population-collapsed data specific to the 0.81 m drop height. Despite observing smaller PFR in NCAA female athletes compared to male athletes for the 0.51 m (NCAA females = 13.5 ± 6.4 N × kg^−1^; NCAA males = 17.6 ± 8.8 N × kg^−1^) and 0.66 m (NCAA females = 21.7 ± 5.3 N × kg^−1^; NCAA males = 22.7 ± 11.4 N × kg^−1^) drop heights, a non-significant increase in PFR for NCAA female athletes was observed for the 0.81 m (NCAA females = 34.1 ± 9.8 N × kg^−1^; NCAA males = 29.7 ± 12.2 N × kg^−1^) drop height, which likely influenced this finding.

Although it is relatively easy to estimate PFR from GRF data, this variable has not been extensively studied in the DJ literature. In the current investigation, PFR showed moderate association with k_v_ and strong association with peak GRF. Although peak GRF and k_v_ are more commonly included as outcome measures in the literature, it is worth noting that concerns have been raised about the validity of k_v_, due to its estimation from linear spring-mass modeling methods [96]. Research has explored PFR in the context of neuromuscular fatigue [6], indicating that higher PFR values may be indicative of increased fatigue and of alterations in the spinal reflex response to landing impact forces. In addition, PFR may reflect a relaxation of active muscle tension in response to the intensity of landing impact. Given these implications, PFR has a biological basis that makes it worth considering as a variable to include in future DJ investigations. Nevertheless, further research is necessary to fully establish and understand the extent of its usefulness and potential applications.

## 5. Limitations

While the sample size of mixed-sex NCAA Division I basketball players and young adults was sufficient to identify the main effects of sex on RSI scores and JH, it did not provide adequate statistical power to detect significance for ROC curve AUCs that indicated fair discrimination when considering NCAA and young adult populations independently. Moreover, the sample homogeneity of NCAA athletes could affect the generalizability of the findings, as all athletes were from the same athletic department. The cross-sectional design of this investigation limits the ability to establish causal relationships, and does not account for potential changes or acute variability in jumping performance over time. However, the estimates of sample size threshold requirements to detect binary classification of sex may prove valuable in guiding the design of future DJ investigations. It is also important to note that participants performed the DJ as a controlled laboratory movement, and, as a result, the results may not fully reflect the complexities of jumping and landing movements performed during sports activities.

## 6. Conclusions

The results revealed significant differences in DJ performance between males and females, with males achieving higher RSI scores and JHs. Interestingly, male NCAA athletes exhibited longer GCTs and slower amortization times compared to female NCAA athletes and young adult males. The reasons for prolonged GCTs in male NCAA athletes remain unclear, but anthropometric factors such as height and limb length may play a role. From ROC curve analysis, RSI scores returned fair to good discrimination between male and female young adults and excellent discrimination between male and female NCAA athletes at specific drop heights. The discriminatory ability of RSI scores varied across different drop heights and populations. These findings highlight the influence of population characteristics and drop height on sex-based differences in DJ GRF measures. In addition to RSI scores and JH, the study highlighted the potential relevance of PFR as a variable to include in future DJ investigations. This study also examined the effects of drop height on DJ GRF measures and identified differences in peak GRF, RFD, and PFR. These findings suggest that drop height influences biomechanical and neuromuscular activation measures. However, the ideal drop height for differentiating between male and female DJ GRF measures remains unclear, and warrants further investigation. Overall, this study addresses several gaps in the existing literature on DJ performance and provides direction for the design of future cross-sectional and longitudinal DJ investigations.

## Figures and Tables

**Table 1:** Participant Characteristics.

	Male		Female	
	NCAA	YA	NCAA	YA
*n*	10	13	11	13
Age (years)	20.1 (1.3)	23.9 (1.7)	19.6 (0.8)	23.3 (1.8)
Body mass (kg)	91.6 (11.8)	80.2 (12.5)	74.4 (10.3)	68.0 (14.5)
Height (cm)	196.9 (8.0)	177.5 (8.4)	181.0 (8.3)	167.3 (8.6)

Values are reported as mean (SD). NCAA = National Collegiate Athletic Association Division I basketball players. YA = physically active young adults.

**Table 2:** Bivariate correlation matrix.

	RSI	JH	GCT	Peak GRF	PFR	RFD	Amort	k_v_
RSI	1.00	0.82 *	−0.51 *	0.08	−0.24	0.36 *	−0.50 *	0.15
JH (m)	-	1.00	−0.01	−0.21 *	−0.24 *	−0.11	0.05	−0.02
GCT (s)	-	-	1.00	−0.37 *	0.13	−0.78 *	0.95 *	−0.20 *
Peak GRF (N × kg^−1^)	-	-	-	1.00	0.66 *	0.81 *	−0.35 *	0.81 *
PFR (N × kg^−1^)	-	-	-	-	1.00	0.33 *	0.17 *	0.51 *
RFD (N × s^−1^ × kg^−1^)	-	-	-	-	-	1.00	−0.74 *	0.59 *
Amort (s)	-	-	-	-	-	-	1.00	−0.17 *
k_v_ (BW × ht^−1^)	-	-	-	-	-	-	-	1.00

* *p* < 0.05. RSI = reactive strength index; JH = jump height; GCT = ground contact time; GRF = vertical ground reaction force; PFR = peak force reduction; RFD = rate of force development; Amort = amortization time; k_v_ = vertical stiffness.

**Table 3:** Post hoc comparisons on significant sex × population interactions.

	NCAA Male	NCAA Female	YA Male	YA Female
GCT (s)	0.45 (0.13)	0.37 (0.07) *	0.39 (0.10)	0.42 (0.10)
PFR (N × kg^−1^)	23.4 11.7)	23.1 (11.2)	16.7 (10.0)	27.0 (14.0) ^Ұ^
Amort (s)	0.24 (0.08)	0.19 (0.04) *	0.20 (0.05) *	0.21 (0.06)

Values are reported as mean (SD). ^Ұ^ significantly different from male physically active young adults (YA; *p* < 0.05). * significantly different from male National Collegiate Athletic Association (NCAA) Division I basketball players (*p* < 0.005). GCT = ground contact time; PFR = peak force reduction; Amort = amortization time.

**Table 4:** Central tendency and dispersion results collapsed across drop height.

	Male	Female	NCAA	Young Adult
RSI	1.11 (0.56)	0.79 (0.29) ^Ұ^	1.00 (0.40)	0.92 (0.54)
JH (m)	0.43 (0.17)	0.31 (0.09) ^Ұ^	0.40 (0.12)	0.35 (0.16) *
GCT (s)	0.43 (0.15)	0.42 (0.12)	0.43 (0.12)	0.42 (0.14)
Peak GRF (N × kg^−1^)	47.2 (12.7)	51.0 (12.2)	48.4 (13.0)	49.6 (12.3)
PFR (N × kg^−1^)	19.3 (11.1)	24.1 (12.8) ^Ұ^	23.2 (11.3)	21.5 (13.0)
RFD (N × s^−1^ × kg^−1^)	123.6 (51.7)	136.3 (52.5)	129.2 (59.5)	130.0 (46.5)
Amort (s)	55.5 (23.9)	54.0 (16.9)	53.8 (20.1)	55.4 (21.1)
k_v_ (BW × ht^−1^)	0.22 (0.09)	0.22 (0.08)	0.23 (0.10)	0.21 (0.07)

Values are reported as mean (SD). ^Ұ^ significantly different from male (*p* < 0.05). * significantly different from National Collegiate Athletic Association (NCAA) Division I basketball players (*p* < 0.05). RSI = reactive strength index; JH = jump height; GCT = ground contact time; GRF = vertical ground reaction force; PFR = peak force reduction; RFD = rate of force development; Amort = amortization time; k_v_ = vertical stiffness.

**Table 5:** Central tendency and dispersion results collapsed across sex and population.

	0.51 m DJ	0.66 m DJ	0.81 m DJ
RSI	0.97 (0.46)	1.05 (0.49)	1.03 (0.52)
JH (m)	0.37 (0.15)	0.39 (0.15)	0.40 (0.16)
GCT (s)	0.41 (0.11)	0.40 (0.11)	0.41 (0.10)
Peak GRF (N × kg^−1^)	39.8 (7.8)	49.4 (9.6) *	58.0 (12.5) * ^Ұ^
PFR (N × kg^−1^)	13.7 (8.0)	21.8 (9.6) *	31.2 (12.0) * ^Ұ^
RFD (N × s^−1^ × kg^−1^)	107.7 (44.0)	132.0 (46.2) *	149.3 (58.1) * ^Ұ^
Amort (s)	0.21 (0.06)	0.21 (0.06)	0.21 (0.06)
k_v_ (BW × ht^−1^)	40.6 (15.3)	55.8 (18.6) *	67.8 (18.6) * ^Ұ^

Values are reported as mean (SD). ^Ұ^ significantly different from 0.66 m depth jump (DJ; *p* < 0.05). * significantly different from 0.51 m DJ) (*p* < 0.05). RSI = reactive strength index; JH = jump height; GCT = ground contact time; GRF = vertical ground reaction force; PFR = peak force reduction; RFD = rate of force development; Amort = amortization time; k_v_ = vertical stiffness.

**Table 6:** ROC curve analysis collapsed across population (*n* = 46).

Variable	0.51m DJ		0.66m DJ		0.81m DJ	
	AUC (*p*)	*n*	AUC (*p*)	*n*	AUC (*p*)	*n*
RSI (m × s^−1^)	0.743 * (0.030)	42	0.639 (0.397)	128	0.732 * (0.045)	46
JH (m)	0.800 * (0.004)	26	0.747 * (0.028)	40	0.843 * (<0.001)	20
GCT (s)	0.531 (1.0)	2554	0.560 (1.0)	702	0.565 (1.0)	584
Peak GRF (N × kg^−1^)	0.578 (0.914)	404	0.628 (0.479)	152	0.667 (0.231)	88
PFR (N × kg^−1^)	0.590 (0.806)	308	0.6862 (0.152)	72	0.7316 * (0.045)	46
RFD (N × s^−1^ × kg^−1^)	0.584 (0.860)	352	0.626 (0.493)	158	0.5841 (0.860)	352
k_v_ (BW × ht^−1^)	0.556 (1.0)	798	0.567 (1.0)	552	0.512 (1.0)	16,436
Amort (s)	0.534 (1.0)	2150	0.555 (1.0)	828	0.548 (1.0)	1070

* significant area under the curve (AUC; *p* < 0.05). *n* = minimum sample size to achieve statistical significance (*p* < 0.05); DJ = depth jump; RSI = reactive strength index; JH = jump height; GCT = ground contact time; GRF = vertical ground reaction force; PFR = peak force reduction; RFD = rate of force development; Amort = amortization time; k_v_ = vertical stiffness.

**Table 7:** ROC curve analysis on NCAA DI basketball athletes (*n* = 20).

Variable	0.51m DJ		0.66m DJ		0.81m DJ	
	AUC (*p*)	*n*	AUC (*p*)	*n*	AUC (*p*)	*n*
RSI (m × s^−1^)	0.687 (0.503)	71	0.515 (1.0)	10,869	0.677 (0.558)	80
JH (m)	0.808 (0.085)	25	0.707 (0.401)	58	0.980 * (<0.001)	7
GCT (s)	0.682 (0.530)	76	0.763 (0.188)	36	0.737 (0.272)	44
Peak GRF (N × kg^−1^)	0.616 (0.929)	186	0.566 (1.0)	582	0.727 (0.312)	48
PFR (N × kg^−1^)	0.636 (0.800)	135	0.576 (1.0)	437	0.606 (0.995)	222
RFD (N × s^−1^ × kg^−1^)	0.727 (0.312)	48	0.768 (0.173)	34	0.788 (0.123)	29
k_v_ (BW × ht^−1^)	0.525 (1.0)	3923	0.626 (0.864)	157	0.576 (1.0)	437
Amort (s)	0.687 (0.503)	71	0.737 (0.272)	44	0.748 (0.236)	40

* significant area under the curve (AUC; *p* < 0.05). *n* = minimum sample size to achieve statistical significance (*p* < 0.05); DJ = depth jump; RSI = reactive strength index; JH = jump height; GCT = ground contact time; GRF = vertical ground reaction force; PFR = peak force reduction; RFD = rate of force development; Amort = amortization time; k_v_ = vertical stiffness.

**Table 8:** ROC curve analysis on young adults (*n* = 26).

Variable	0.51m DJ		0.66m DJ		0.81m DJ	
	AUC (*p*)	*n*	AUC (*p*)	*n*	AUC (*p*)	*n*
RSI (m × s^−1^)	0.810 * (0.039)	25	0.744 (0.156)	41	0.774 (0.087)	32
JH (m)	0.827 * (0.024)	22	0.810 * (0.039)	25	0.827 * (0.024)	22
GCT (s)	0.604 (0.915)	231	0.560 (1.0)	706	0.554 (1.0)	870
Peak GRF (N × kg^−1^)	0.560 (1.0)	706	0.691 (0.364)	69	0.667 (0.494)	90
PFR (N × kg^−1^)	0.720 (0.234)	51	0.821 * (0.028)	23	0.804 * (0.045)	26
RFD (N × s^−1^ × kg^−1^)	0.536 (1.0)	1961	0.560 (1.0)	706	0.583 (1.0)	360
k_v_ (BW × ht^−1^)	0.554 (1.0)	870	0.506 (1.0)	69,451	0.548 (1.0)	1104
Amort (s)	0.586 (1.0)	337	0.583 (1.0)	360	0.587 (1.0)	334

* significant area under the curve (AUC; *p* < 0.05). *n* = minimum sample size to achieve statistical significance (*p* < 0.05); DJ = depth jump; RSI = reactive strength index; JH = jump height; GCT = ground contact time; GRF = vertical ground reaction force; PFR = peak force reduction; RFD = rate of force development; Amort = amortization time; k_v_ = vertical stiffness.

## Data Availability

The data presented in this study are available on request from the corresponding author. The data are not publicly available due to privacy restrictions.
